# Electrocortical therapy for motion sickness

**DOI:** 10.1212/WNL.0000000000001989

**Published:** 2015-10-06

**Authors:** Qadeer Arshad, Niccolo Cerchiai, Usman Goga, Yuliya Nigmatullina, R. Ed Roberts, Augusto P. Casani, John F. Golding, Michael A. Gresty, Adolfo M. Bronstein

**Affiliations:** From Imperial College (Q.A., N.C., U.G., Y.N., R.E.R., M.A.G., A.M.B.), Charing Cross Hospital, London, UK; Otorinolaringoiatria 1 Universitaria (N.C., A.P.C.), Pisa, Italy; and the University of Westminster (J.F.G.), London, UK.

Given a sufficiently provocative stimulus, almost everyone can be made motion sick, with approximately one-third experiencing significant symptoms on long bus trips, on ships, or in light aircraft.^1–4^ Current countermeasures are either behavioral or pharmacologic. Behavioral measures include habituation/desensitization treatment protocols^5^ as well as positioning the head in alignment with the direction of the gravito-inertial force and maintaining a stable horizontal reference frame.^5^ Pharmacologic measures include antimuscarinics, H1 antihistamines, and sympathomimetics, which all detrimentally impact upon cognitive function, rendering them inappropriate for occupational use.^5^ All current therapies are only partially effective.

Since a functioning vestibular system is critical to the development of motion sickness,^1^ we proposed that suppressing vestibular activity could increase tolerance to nauseogenic motion stimuli. We previously showed that application of transcranial direct current stimulation (tDCS), specifically unipolar cathodal stimulation over the left parietal cortex, results in suppression of the vestibular system.^6^ Herein, we assessed whether such suppression of vestibular activity using tDCS in normal controls may alleviate motion sickness.

## Classification of evidence.

This study provides Class II evidence that in normal volunteers undergoing off-axis rotation, left parietal cortex cathodal stimulation increases the time to the development of moderate nausea.

## Methods.

We implemented a well-established model of inducing motion sickness termed off-vertical axis rotation (OVAR). Subjects were seated in a motorized chair (NKI; Pittsburgh, PA) with the torso restrained by a 5-point seatbelt with the head, legs, and feet additionally secured with cushioned clamps. The chair was rotated rightwards in the dark, accelerating and tilting gradually over 30 seconds to reach a constant rotational velocity of 72 deg/s, frequency of 0.2 Hz, at a tilt of 18 degrees.^7^ Twenty right-handed subjects (10 M; 10 F not within 3 days of menstruation) were randomly allocated into 2 age- and sex-matched groups (groups 1 and 2). It was ensured that individuals in both groups were matched for susceptibility to motion sickness as determined by adult-based motion sickness questionnaire scores (MSB scores; see the figure, B for each individual's susceptibility score).^8^

**Figure:**
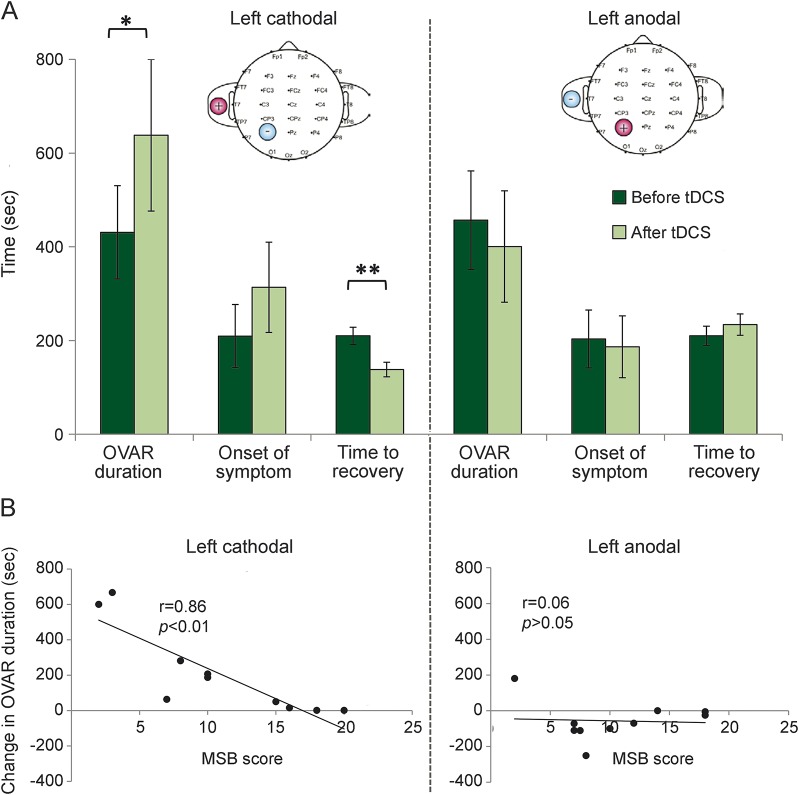
Effects of electrocortical stimulation on motion sickness susceptibility (A) (Left upper panel) Following left cathodal transcranial direct current stimulation (tDCS) stimulation, the susceptibility to motion sickness was reduced, as reflected by the significant increase in off-vertical axis rotation (OVAR) duration required to induce moderate nausea. Also, we observe a significant reduction in the time taken for symptom recovery. In contrast, following left anodal stimulation, we did not observe any significant effects (right upper panel). (B) Adult motion sickness susceptibility scores (MSB) showed significant correlation with the change in OVAR duration (post-pre tDCS) for the left cathodal stimulation only (left lower panel), indicating that less susceptible subjects derived the largest benefit from tDCS. Error bars represent standard errors. *p < 0.05; **p < 0.01.

The experimental design was specifically chosen to test whether tDCS is potentially effective as a prophylactic or abortive treatment. Both groups underwent an initial OVAR session during sham tDCS stimulation only. The time taken to self-report (1) onset of symptoms (i.e., stomach awareness); (2) the primary outcome measure, onset of moderate nausea (i.e., total OVAR duration, with no upper time limit); and (3) subsequent self-recovery were recorded. Subjects were given a 1-hour rest period in a separate room, after which all reported full recovery. Critically, despite recovery, at this time point previous research has shown that subjects have higher motion sickness susceptibility,^9^ thus allowing us to test the efficacy of tDCS during enhanced susceptibility. After recovery, unipolar tDCS was applied (1.5 mA with a ramp-up and fade-out time of 10 seconds; electrode placement area 25 cm^2^; for electrode placement on the scalp, we parted the hair and electrodes were held in place with an EEG cap^6^) with 2 possible different polarities in a double-blind design.^6^ For group 1 (cathodal) and group 2 (anodal), stimulation was applied initially for 15 minutes immediately prior to the second OVAR session over left parietal cortex and stimulation continued for either a further 15 minutes during the rotation or until subjects reported moderate nausea (i.e., whichever came first). No subjects dropped out or complained of any symptoms associated with either tDCS stimulation or tDCS-mediated vestibular suppression.

## Results.

The figure summarizes the results of the experiment. Repeated-measures analysis of variance for cathodal tDCS stimulation with within-subjects factors measurement (OVAR duration, first onset of symptoms, and time to recovery) and condition (before tDCS, after tDCS) showed a significant measurement × condition interaction (*F* = 9.48, *df* = 2, *p* = 0.033; figure, A). Post hoc paired *t* tests (2-tailed) with Bonferroni corrections showed that following cathodal stimulation, OVAR duration (i.e., time taken for the onset of moderate nausea) was significantly increased (*t* = 2.68, *p* < 0.05; figure, A). In contrast, for anodal stimulation, the measurement × condition interaction was not significant. That is, following left cathodal stimulation, it took subjects in the second OVAR session 207 seconds (range 32–382 seconds) longer to develop moderate nausea, whereas following left anodal stimulation subjects developed moderate nausea on average 57 seconds sooner (range 22 to −153 seconds). Notably, those subjects less susceptible to motion sickness (i.e., lower MSB scores) derived the largest benefit following left cathodal tDCS stimulation (*r* = 0.86, *p* < 0.001; figure, B). Further, the time taken to recover following cathodal stimulation was significantly reduced (*t* = 6.0, *p* < 0.001; figure, A).

## Discussion.

Following cathodal tDCS over the left hemisphere, we observed both an increased duration in the time taken to develop moderate nausea during OVAR and a more rapid recovery from symptoms. As no significant effects were observed during anodal stimulation, this excludes the role of both adaptation and nonspecific effects due to tDCS.^10^ We provide a novel treatment for motion sickness that is, so far, apparently free of side effects.
